# High-Performance Series-Fed Array Multiple-Input Multiple-Output Antenna for Millimeter-Wave 5G Networks

**DOI:** 10.3390/s25041036

**Published:** 2025-02-09

**Authors:** Nabeel Alsaab, Khaled Alhassoon, Fahd Alsaleem, Fahad Nasser Alsunaydih, Sayed O. Madbouly, Sherif A. Khaleel, Allam M. Ameen, Mahmoud Shaban

**Affiliations:** 1 Department of Electrical Engineering, College of Engineering, Qassim University, Buraydah 52571, Saudi Arabia; k.hassoon@qu.edu.sa (K.A.); f.alsaleem@qu.edu.sa (F.A.); f.alsunaydih@qu.edu.sa (F.N.A.); so.ossman@qu.edu.sa (S.O.M.); 2 College of Engineering and Technology, Arab Academy for Science, Technology and Maritime Transport, Aswan 81511, Egypt; sherif.abdalla@aast.edu; 3 Microstrip Department, Electronics Research Institute, New Nozha, Cairo 11843, Egypt; allamameen@eri.sci.eg

**Keywords:** millimeter wave (mmWave), antennas, MIMO systems, high isolation, 5G communications

## Abstract

This research presents a high-performance design for a multiple-input multiple-output (MIMO) antenna intended for operation within the 28 GHz band. The four-port MIMO antenna configuration, featuring 1 × 8 series-fed arrays for each port, has demonstrated peak gains of 15.5 dBi and bandwidths of 2 GHz. This improved antenna performance results from carefully optimized antenna spacing and a decoupling approach involving well-designed metamaterial cells, effectively minimizing interference between antenna elements. The system exhibits remarkably low mutual coupling, measuring below −40 dB, with envelope correlation coefficients of 0.00010, diversity gains nearing 10 dB, and a channel loss capacity of 0.11 bit/s/Hz across the frequency spectrum under investigation. Experimental evaluations have confirmed these improvements, establishing the proposed design as a robust candidate suitable for a wide range of millimeter-wave communication systems.

## 1. Introduction

Recently, communication systems have come under great pressure from an increasing number of users adopting electronic devices. It is understood that higher data rates are demanded, and great development and integration with wireless technology and broadband are necessary to achieve those speeds. Accordingly, with each new generation of wireless technology, new features are being launched, along with better advantages and overall performance compared to the previous generation. Fifth-generation (5G) wireless technology employs a mix of different frequency bands, each having its unique characteristics. For outdoor users, sub-1 GHz bands have large coverage areas, while mid-band frequencies (2.5, 3.5, 3.7–4.2, and 4.8–5 GHz) provide a balance between coverage and capacity. On the contrary, the millimeter-wave (mmWave) bands, which operate above 24 GHz, will produce a very high data rate, but they have limited coverage at present. Moreover, the Federal Communications Commission (FCC) allocated some bands for 5G, including 24 GHz (N257 and N261), 28 GHz (N261), 37–40 GHz (N260 and N261), and 47 GHz for licensed use [1,2,3,4,5]. The 64–71 GHz band is also available for unlicensed wireless communication and is the frequency range for Wi-Gig (Wireless and Gigabit) and other high-speed wireless systems [6,7].

Multiple-input multiple-output (MIMO) antenna technology utilizes multiple antennas at both the transmitter and receiver to improve communication performance across various dimensions. The use of MIMO systems enables the simultaneous transmission and reception of multiple data streams, thereby increasing data throughput, enhancing signal quality, and improving overall wireless communication efficiency. Consequently, the exploration of MIMO antenna design for 5G, particularly in the millimeter-wave (mmWave) frequency range, presents a promising and compelling area for extensive research [8,9,10]. The most critical requirements from the point of view of MIMO antenna design are maximizing spectral efficiency, improving interference mitigation, and optimizing bandwidth adaptation—all essential needs in any 5G technology. Thus, depending on network demand, they require spectrum-efficient value chains, effective interference management, and dynamic bandwidth allocation. MIMO antennas have long been considered for maximizing spectral efficiency using spatial multiplexing and beamforming to exploit the advantages of such adaptively planned radiation patterns, polarization diversity, and directional beamforming techniques to reduce radiation interference [11,12,13,14,15].

The main challenges in MIMO antenna design are minimizing mutual coupling between antenna elements, reducing the size and shape of antennas, maximizing antenna gains, increasing the bandwidth and frequency of use, and ensuring a high antenna efficiency. Therefore, different antenna designs have been suggested in previous research as possible solutions for alleviating the stated challenges and facilitating the MIMO antenna development process.

The study presented in reference [16] introduced a MIMO antenna structure designed for 5G applications, operating at a frequency of 28 GHz. The design features three circular rings enclosed within an infinity-shaped shell aimed at enhancing the antenna’s performance for high-frequency communication. Simulations were shown to give a gain of 6.1 dBi and an antenna efficiency of 92%. A compact planar MIMO antenna configuration featuring a tree-like structure, as outlined in reference [17], was designed to support a wide bandwidth suitable for 5G applications. It is composed of four curved elements to cater for wideband performance. Radiation patterns were studied at 28 GHz, 33 GHz, and 38 GHz, with the maximum total gains realized being 10.58 dB, 8.87 dB, and 11.45 dB, respectively. In [18], a dual-band MIMO antenna with high gain operates mainly at 28 and 38 GHz with peak realized gains at 4.15 dBi and 7.73 dBi and total efficiencies of 80.13% and 85.44%, respectively. The crescent-shaped slot elliptical MIMO antenna defined in [19] operates at dual frequencies of 28/38 GHz, which utilizes a 1 × 4 linear array with a peak gain of 10.4 dBi and a bandwidth of 7.8 GHz. The implementation in [20] attains isolation of the elements in a MIMO antenna up to 23 dB using spatial and polarization diversity. The constructed design is in the frequency bands of 27.25 to 29 GHz and 34.5 to 41 GHz and gains of 9.5 dBi in 28 GHz and 11.7 dBi in 38 GHz.

Metamaterials, engineered materials with distinctive electromagnetic properties, are crucial in enhancing gain and isolation in MIMO systems. They improve gain by efficiently focusing electromagnetic waves, which enhances directivity and radiated power. Furthermore, metamaterial structures create barriers that absorb or block unwanted radiation between closely spaced antennas, thereby improving isolation. This isolation is essential for preventing interference and crosstalk in densely packed MIMO configurations. By strategically integrating metamaterials, the properties of antennas can be tailored to optimize performance, increasing gain and strengthening isolation in MIMO systems, ultimately enhancing communication reliability and efficiency [21,22,23]. Studies have been performed on improving isolation in dense arrays of antennas using periodic metamaterial photonic bandgap structures to approach applications such as MIMO systems and Synthetic-Aperture Radar (SAR) for isolation problems [24]. Further investigations aimed to understand the envelope of surface wave effects due to antenna arrays that involve mostly visible contributions from metasurface inclusions in MIMO and SAR systems [25]. Other methodologies use metamaterials as electromagnetic bandgap (EM-BG) decoupling slabs to reduce mutual coupling in antenna arrays, enhancing output in harsh operating environments of communication [26,27]. The research presented in [28] explored achieving mutual-coupling isolation in densely packed array antennas using an embedded metamaterial EM bandgap decoupling slab. Regarding MIMO antenna compactness, a compact four-element MIMO antenna customized for 5G millimeter-wave applications in the 37–39 GHz frequency range has been introduced in reference [29]. Additionally, reconfigurable MIMO structures operating at 28/38 GHz with wideband coverage and high isolation for advanced mmWave applications has been reported in [30]. More recently, another study investigated the design of a reconfigurable MIMO antenna, aiming to enhance MIMO performance in automotive 24 GHz radar sensors [31].

The proposed design here is of substantial significance for several key reasons:

− It achieves peak gains of 15.5 dBi at 28 GHz, enhancing signal directivity and extending communication range.
− The antenna supports a bandwidth of 2.0 GHz, exhibiting a low reflection coefficient of −35 dB, facilitating low-loss operation.
− It demonstrates more than 40 dB isolation levels across its ports, effectively rejecting interference and ensuring robust, clear signal transmission.
− Experimental measurements have confirmed the achieved results, emphasizing the design’s reliability and positioning it as a promising option for future 5G millimeter-wave wireless applications.

## 2. Materials and Methods

As per the transmission-line model, a microstrip antenna is conceptualized with two radiating slots separated by a low-impedance transmission line characterized by dimensions such as length (L), impedance (Z_o_), width (W), and the height of the antenna substrate (h), ensuring that the length over height ratio (L/h) is significantly greater than 1. The radiated fields extend beyond the physical edges of the rectangular patch, enhancing the antenna’s overall performance. The fringing effect, which extends beyond the patch’s boundaries, is influenced by the patch size, substrate height, and dielectric constant. Although larger L/h ratios can reduce the impact of fringing, electromagnetic fields still induce slight deviations in the antenna’s resonant frequency (f_r_). The design process commences with determining the dimensions of the patch array. The subsequent set of equations elucidates this calculation. The effective relative permittivity for the substrate can be calculated as per the following formula [32]:

(1) $$\varepsilon_{\mathrm{reff}}=\frac{\varepsilon_{r}+1}{2}+\frac{\varepsilon_{r}-1}{2}{\left[1+12\frac{h}{W}\right]}^{\frac{-1}{2}}$$

The patch effective length, denoted as L_eff_, is determined by:

(2) $$\Delta L=0.412h\frac{\left(\varepsilon_{\mathrm{reff}}+0.3\right)\left(\frac{W}{h}+0.264\right)}{\left(\varepsilon_{\mathrm{reff}}-0.254\right)\left(\frac{W}{h}+0.8\right)}$$

The width of a microstrip patch antenna is related to its resonant frequency and the effective dielectric constant of the substrate material. The relationship is derived from the antenna’s design and is crucial for determining its operating frequency. The patch width, W, is given by the formula:

(3) $$W=\frac{c}{2f_{r}}\sqrt{\frac{2}{\varepsilon_{r}+1}}$$

Due to the field fringing effect, the patch length is extended by ΔL on both sides. The effective length L_eff_ is given by:

(4) $$L_{\mathrm{eff}}=L+2\Delta L$$

where L represents the original length of the patch. Thus, the patch length can be calculated as:

(5) $$L=\frac{c}{2f_{r}\sqrt{\varepsilon_{\mathrm{reff}}}}-2\Delta L$$

At resonance, the input resistance at the edge of the patch (y = 0) can be accurately characterized by the expression:

(6) $$R_{\mathrm{in}}\left(0\right)=\frac{1}{2\left(G_{1}+G_{12}\right)}$$

where G_1_ represents the conductance of a single slot of a rectangular patch that can also be obtained by using the field expression derived by the cavity model, and G_12_ accounts for the conductance induced by mutual effects between the two radiating slots of the patch. The conductance terms G_1_ and G_12_ play crucial roles in determining the overall performance and efficiency of the microstrip patch antenna system, and they can be calculated using:

(7) $$G_{1}=\frac{1}{120\pi^{2}}\overset{\pi }{\underset{0}{\int }}{\left[\frac{\sin\left(\frac{k_{0}w}{2}\cos\theta \right)}{\cos\theta }\right]}^{2}\sin^{3}\theta \;d\theta$$

(8) $$G_{12}=\frac{1}{120\pi^{2}}\overset{\pi }{\underset{0}{\int }}{\left[\frac{\sin\left(\frac{k_{0}w}{2}\cos\theta \right)}{\cos\theta }\right]}^{2}J_{0}\left(K_{0}L\sin\theta \right)\sin^{3}\theta d\theta$$

where J_0_ is the Bessel function of the first kind of order zero. K_0_ is free-space phase constant that is equal to 2π/λ_0_, where λ_0_ is free-space wavelength.

The input impedance of the patch at a distance y_0_, namely inset length (L_inset_) from the patch edge along its length, is as follows:

(9) $$R_{\mathrm{in}}\left(y_{0}\right)=R_{\mathrm{in}}\left(0\right)\cos^{2}\left(\frac{\pi }{L}y_{0}\right)$$

Many important design parameters and equations must be considered when making a series-fed antenna array. The distance of separation of antenna elements plays a vital role in obtaining the formulation intended in the radiation pattern. For instance, in a uniform linear array, the spacing distance (L_f_) is generally set at half-wavelength (λg/2) to encourage constructive interference along the main beam direction to facilitate effective beamforming. The array factor explains how the individual elements collaborate with each other to form the complete production of the radiation pattern of the array. For a uniform linear array with N elements, the array factor (AF) can be expressed as:

(10) $$\mathrm{AF}=\overset{N}{\underset{1}{\sum }}e^{j\left(n-1\right)\mathrm{kdcos}\left(\theta \right)}$$

The array factor (AF) is calculated as a function of off-broadside angles, as the contribution of each element to the overall antenna radiation pattern is expressed. It is good to have an impedance match in an efficient antenna array. Good impedance matching makes an efficient transfer of power inside the array. The antenna elements and feed networks should be impedance matched to system impedance (say 50 Ω) to reflect the minimum and increase overall performance.

MIMO antenna assessment parameters gauge performance. One of the significant quantifications is the envelope correlation coefficient (ECC), which measures how the signal envelopes received through different antennas correlate with one another. Lower ECC would mean stronger diversity performance because it can mean better reception of uncorrelated signals and better diversity gains. Diversity gain (DG) refers to the improvement in signal reception quality obtained by using multiple antennas to counteract signal fading through spatial diversity.

Another critical parameter is the “mean effective gain”, which describes the power gain of antenna pattern averaged over all the angles in a given scenario, thus giving an idea about the overall capability of the system itself. Combining the Effective Diversity Gain incorporates part diversity gain through antenna correlation, providing better insight into system diversity performance. Channel Capacity Loss (CCL) defines the amount by which the maximum potential data rate is reduced due to interference or noise and provides figures to measure the channel-inherent inefficiencies affecting capacity.

In this case, the usefulness of ECC computation is related to signal correlation and its effects on system performance. ECC can be calculated in different ways depending on the purpose.

(11) $$\mathrm{ECC}=\frac{{\left|S_{\mathrm{ii}}^{*}S_{\mathrm{ij}}+S_{\mathrm{ji}}^{*}S_{\mathrm{jj}}\right|}^{2}}{\left(1-\left({\left|S_{\mathrm{ii}}\right|}^{2}+{\left|S_{\mathrm{ji}}\right|}^{2}\right)\right)+\left(1-\left({\left|S_{\mathrm{jj}}\right|}^{2}+{\left|S_{\mathrm{ij}}\right|}^{2}\right)\right)}$$

where the terms S_ii_, S_jj,_ S_ii_*, and S_ji_* refer to the complex and conjugate complex elements of the scattering matrix, demonstrating signals received by antennas i and j, respectively.

ECC directly affects the DG, measured in dB, as follows:

(12) $$\mathrm{DG}=10\sqrt{1-{\left(\mathrm{ECC}\right)}^{2}}$$

The CCL metric quantifies the decrease in the highest attainable data rate by considering the logarithm of the magnitude of the received signal coefficients. Thus, it is computed by:

(13) $$\mathrm{CCL}=-\log_{2}\left|\beta^{R}\right|$$

where

$$\beta^{R}=\left(\begin{array}{cccc} \beta_{11} & \beta_{12} & & \\ & & \cdots & \beta_{1N}\\ \beta_{21} & \beta_{22}\\ \vdots & \vdots & \cdots & \vdots \\ \beta_{N1} & \beta_{N2} & \cdots & \beta_{NN} \end{array}\right)$$

where

$$\beta_{\mathrm{ii}}=1-\overset{N}{\underset{n=1}{\sum }}\left|S_{\mathrm{in}}^{*}S_{\mathrm{ni}}\right|$$

and

$$\beta_{\mathrm{ij}}=-\overset{N}{\underset{n=1}{\sum }}\left|S_{\mathrm{in}}^{*}S_{\mathrm{nj}}\right|$$

β_R_ characterizes a matrix representative of the received signal coefficients. $\beta_{\mathrm{ii}}$ represents the coefficients corresponding to the power received by antenna ii after considering the powers received from other antennas. $\beta_{\mathrm{ij}}$ represents the coefficients that describe the correlation between the signals received by two antennas i and j, counting i, j from 1 to N, where N is the total number of the MIMO antennas.

## 3. Results and Discussion

**Figure 1:**
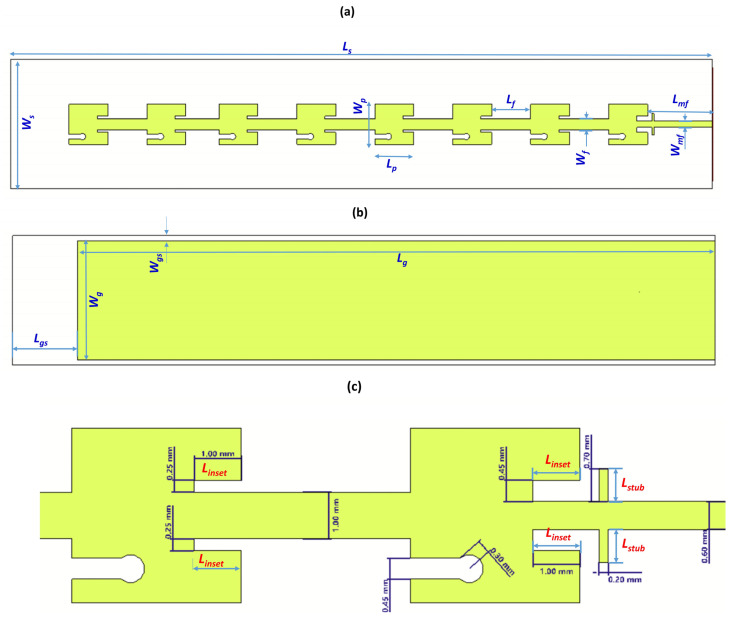
Single-element antenna: (**a**) front view, (**b**) back view, and (**c**) patch slot, inset, and stub dimensions.

Figure 1 depicts the structure of a single port antenna array constructed on a low-loss Rogers RT-5880 substrate with the thickness (h) of 0.787 mm, a dielectric constant (ε_r_) of 2.2, and a loss tangent of 0.0009. This antenna array comprises 1×8 rectangular patch elements fed via a microstrip feedline. Each antenna element consists of a modified single radiating patch with dimensions W_p_ × L_p_. The combination of inset feeders of each dimension, W_inset_ × L_inset_, improves the impedance matching of the antenna. Aimed to enhance the bandwidth of antenna, two rectangular slots, W_slot_ × L_slot_, were plunged at distances W_p_/4 from left and right edge of the patch. The slotted patches were fed through a 50 Ω microstrip feedline, defined by width W_mf_ and length L_mf_. The substrate and ground plane are defined by dimensions W_s_, L_s_ and W_g_, L_g_, respectively. All slot dimensions were carefully adjusted to achieve the best impedance matching at the desired resonant frequency.

Insets in rectangular patch antennas play an important role in adjusting the input impedance of antenna arrays for proper impedance matching. By strategically placing insets within the patch structure, the input impedance is adjusted according to Equation (9). This adjustment is crucial for efficient power transfer from the transmission line to the antenna element, minimizing signal reflections and optimizing performance parameters. In case of insufficient impedance matching, additional stubs with calibrated lengths (L_stub_) and positions can be added to the transmission line feeder to fine-tune the impedance. In this regard, we performed a geometric parametric sweep to optimize the lengths of inset feeders and stubs. From simulation results, it was found that these dimensions are the most influential factor in impacting the input impedance of the antenna array.

**Figure 2:**
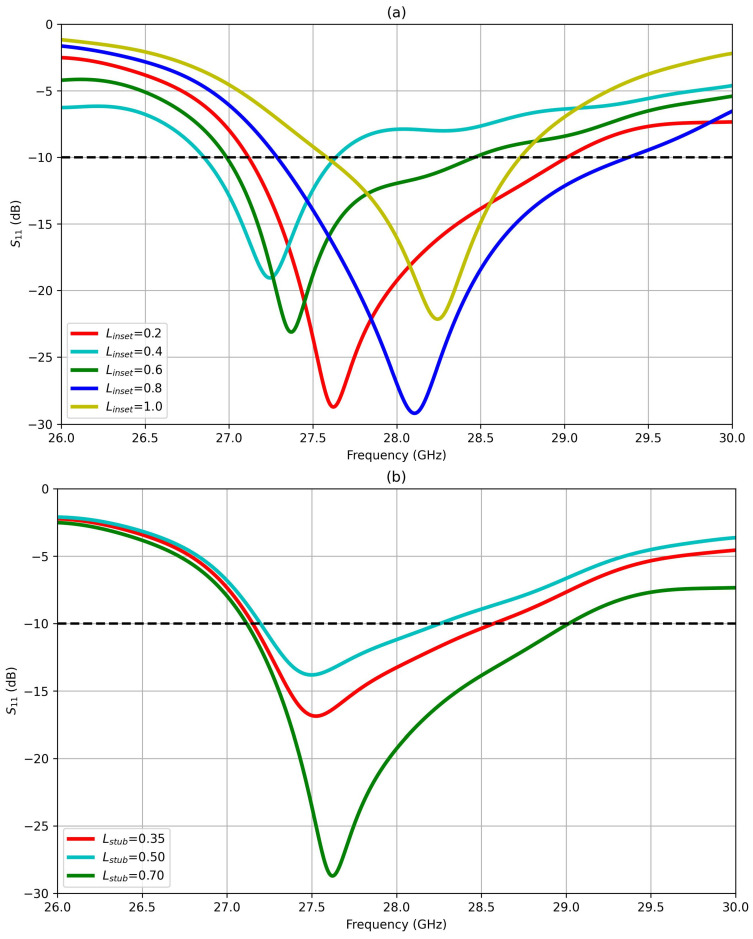
Parametric sweep of (**a**) L_inset_ and (**a**) L_stub_ of proposed antenna design.

Table 1 and Figure 1 offer a detailed overview of all design parameters. The impedance matching was achieved using insets created in the first patch, in addition to two stubs placed before that patch. The insets and stub dimensions were critical for achieving impedance matching and controlling the bandwidth, as indicated in the parametric sweeps of L_inset_ and L_stub_ in Figure 2, where the reflection coefficient S_11_ of the antenna structure was computed.

The parametric sweep analysis reveals that among all the dimensions outlined in Table 1, the inset length (L_inset_ or y_0_) stands out as the most critical parameter. Altering L_inset_ from 0.2 mm to 1.0 mm resulted in significant variations in the resonance frequencies throughout the operating band, as well as in the bandwidth values and reflection coefficients, as illustrated in Figure 2. These changes arise from the intricate interplay between the two inset lengths and the antenna’s input impedance. Adjusting the inset length leads to noticeable shifts in the impedance characteristics, subsequently influencing the resonance behavior, bandwidth efficiency, and reflection properties of the antenna system. Optimal performance was attained with inset and stub lengths set at 0.7 mm.

**Figure 3:**
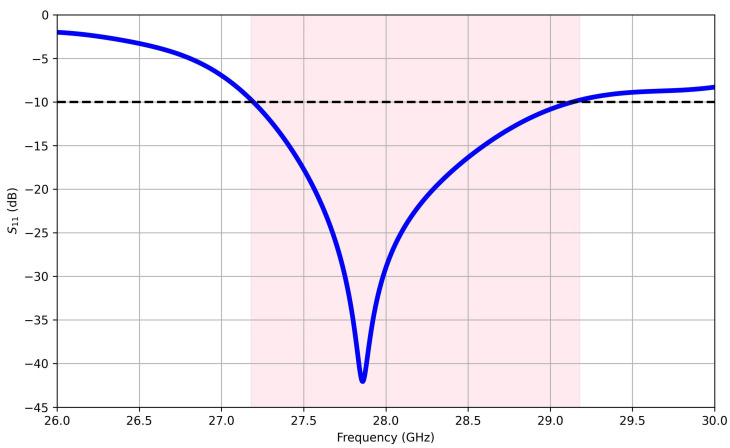
Computed reflection coefficient of single-port antenna versus frequency. The dashed line highlights the −10 dB borderline and the highlighted area represents the bandwidth range, in which the S11 parameter is less than −10 dB.

Optimized low loss with broadening bandwidth was obtained as shown in Figure 3. The antenna’s reflection coefficient, demonstrates resonance at a frequency of 27.85 GHz, corresponding to a reflection coefficient level of −42 dB and a bandwidth extending from 27.2 to 29.2 GHz, which is corresponding to a bandwidth of 2 GHz. These simulated results play a crucial role in forecasting and evaluating the antenna’s performance before the construction of a prototype of the proposed antenna structure. These results were confirmed using measurement methods of the fabricated prototype of the proposed antenna.

**Figure 4:**
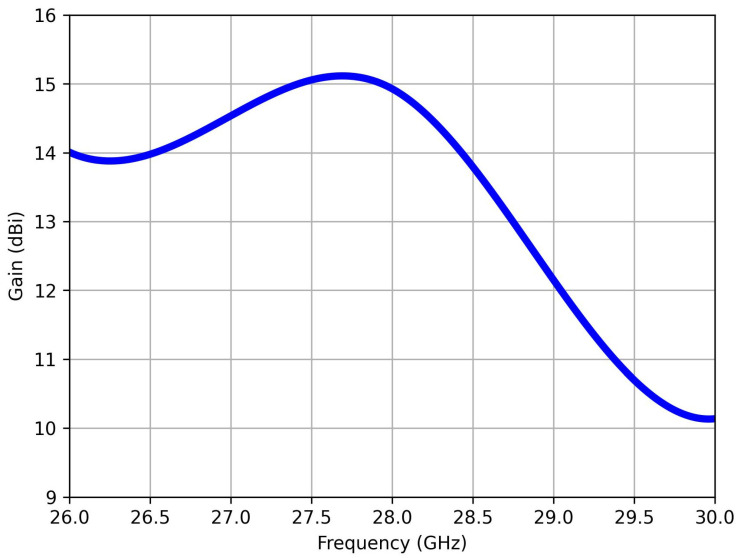
Computed peak gain of single-port antenna versus frequency.

Figure 4 illustrates the computed peak gain of the proposed antenna versus frequency. The 1 × 8 series-fed antenna exhibits a peak gain of approximately 15 dBi at the resonance frequency of 28 GHz. This indicates the antenna’s ability to concentrate the radiating EM energy in a specific direction at the target frequency band, underscoring its suitability for N261 band applications. The peak gain value serves as a testament to the antenna’s ability to transmit and receive signals with heightened effectiveness and precision within the designated frequency range.

### 3.1. Four-Port MIMO Array

**Figure 5:**
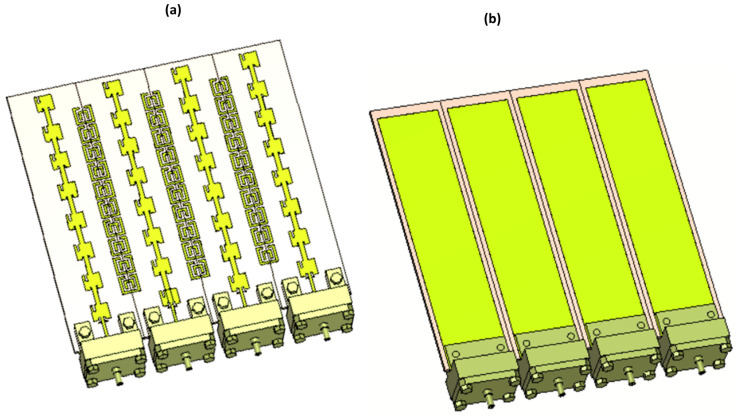
(**a**) Front view and (**b**) back view of 4-Port MIMO antenna.

The MIMO configuration of this design is customized for a four-port MIMO antenna array computed at the resonance frequency of 28.8 GHz. Special attention is paid to the spacing between antenna elements to boost isolation, reduce mutual coupling, and enhance MIMO system performance. This MIMO antenna setup comprises four individual radiating elements compactly arranged within an 80 × 68 mm^2^ area, utilizing the same substrate as the single-array antenna. Figure 5 provides top and bottom views of the MIMO antenna structure, featuring sub-miniature version A (SMA) connectors for each port.

**Figure 6:**
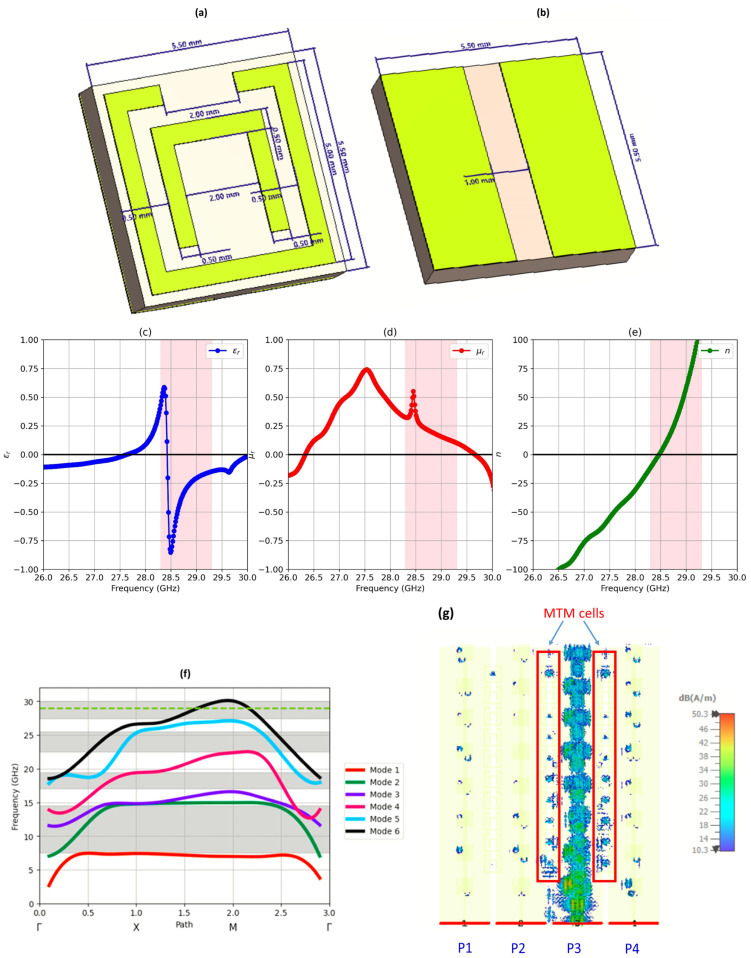
Unit cell MTM in (**a**) top and (**b**) bottom views and its extracted parameters: (**c**) ε_r_, (**d**) µ_r_, (**e**) n. (**f**) Dispersive diagram exhibiting EM bandgaps detected in the unit cell structure and (**g**) current distribution resulting from excitation of port 3.

The achieved significant reduction in mutual coupling due to the key role of the designed MTM cell is depicted in Figure 6. The extracted relative permittivity (ε_r_), relative permeability (µ_r_), and refractive index (n) exhibit both positive and negative values that vary with frequency. Specifically, the design outputs feature negative values of εr across the target frequency range and demonstrate resonant behavior. This underlines the behavior of the cell as an MTM. Regarding gain, the gain is enhanced by the effects of the MTM to focus EM energy in specific directions. Additionally, concerning isolation, it absorbs or prevents surface wave propagation in specific directions. The split-ring-like MTM cells operate similarly to bandstop filters, filtering out unwanted interference and thus improving the attenuation of mutual coupling.

The metamaterial unit cell experienced further investigation through dispersive analysis, as depicted in the frequency dispersion diagram presented in Figure 6f. The eigen-solutions of the electromagnetic wave equation revealed broadband bandgaps evident in the lower-order modes, alongside a narrower bandgap spanning 2.6 GHz within the frequency range from 27.4 to 30 GHz, encompassing the required frequency of 28 GHz. This observation solidifies the role of the unit cell in acting as a bandgap structure, effectively mitigating coupling between MIMO elements within the examined frequency band of interest. Consequently, this boosts isolation capabilities, thereby optimizing the performance of the proposed antenna structure.

Figure 6g presents the surface current distribution of the MIMO structure with excitation of port 3. This demonstrates effectiveness of the implemented metamaterial cells in mitigating surface wave propagation. The MTM cells significantly reduce the impact of mutual coupling between adjacent ports. This reduction in mutual interference enhances the overall performance of the MIMO system by improving signal integrity and minimizing unwanted electromagnetic interactions between antenna elements. The analysis further confirms that the most affected ports are those located closest to the central ports, port 2 and 3, where the surface currents are more concentrated. This suggests that while the metamaterial cells successfully suppressed most of coupling effects, the influence of mutual currents remains more pronounced in specific regions of the structure, particularly near the middle ports. By symmetry, similar results are obtained for excitation of the other ports.

**Figure 7:**
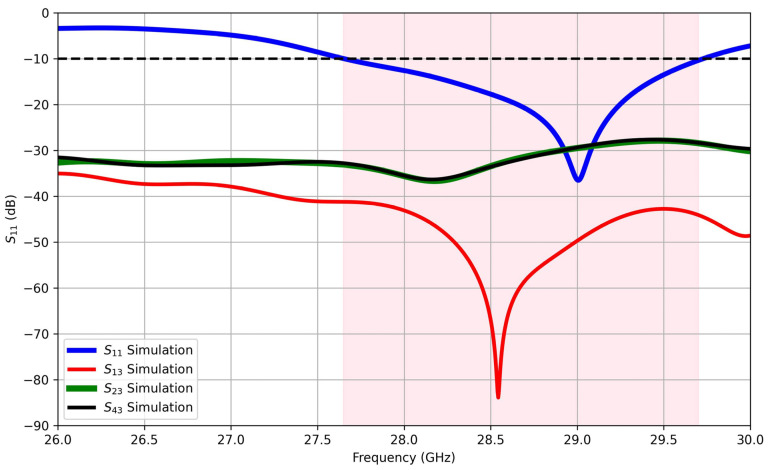
Simulated S-parameters versus frequency of the proposed MIMO antenna.

The simulation results in Figure 7 of the S-parameters exhibit the mutual coupling coefficients (S_13_, S_23_, S_42_) within the MIMO system. Port 2 is specifically selected as the central port significantly influenced by its neighboring ports. The illustrated figure demonstrates that the mutual coupling remains consistently below −41 dB across the entire operational bandwidth. This observation emphasizes the effectiveness of the proposed decoupling methodology, highlighting successful mitigation of interference among the MIMO ports. Consequently, the achieved high isolation among the MIMO ports signifies the effectiveness of the decoupling approach, ensuring enhanced performance and minimized interference in the proposed MIMO structure.

**Figure 8:**
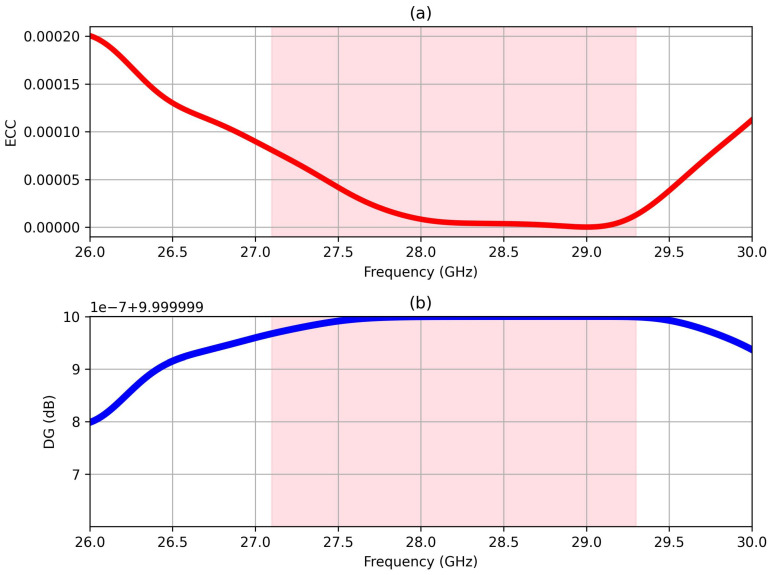
Variation in (**a**) ECC and (**b**) DG of 4-port MIMO antennas versus frequency.

In Figure 8, the graphical representation illustrates the computed frequency-dependent ECC and DG of the MIMO structure. Throughout the frequency spectrum, the ECC values consistently remain below 0.00010, which is much lower than the ECC conventional threshold of 0.5, indicating significant isolation between antenna elements and minimal signal correlation. Simultaneously, the DG approaches the 10 dB, indicating robust signal reception in that direction alongside effective fading mitigation. Moreover, the CCL values demonstrate commendable performance. Specifically, at 28 GHz, the CCL is computed to be 0.11 bit/Hz/s, which is lower than the standard reference of 0.4 bit/Hz/s, signifying a substantial reduction in channel capacity loss and enhanced system efficiency.

**Figure 9:**
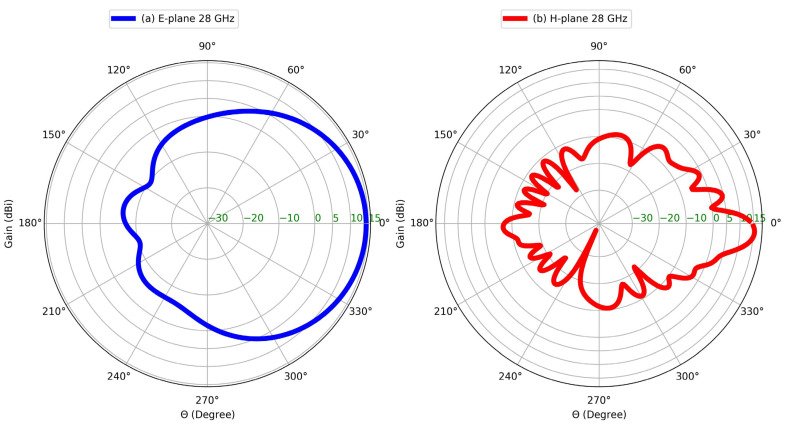
Radiation patterns of 4-port MIMO antenna: (**a**) E-plane, (**b**) H-plane at 28 GHz.

Figure 9 depicts the computed radiation pattern of the MIMO antenna. At 28 GHz and in the E-plane direction, the antenna achieves a gain of 14.4 dBi, with the main lobe pointed at θ = 2° and a beamwidth of 70°. In the H-plane, the main lobe exhibits a gain of 15.5 dBi, directed at θ = 3°, with a beamwidth of 10.4°. This emphasizes the directional property of the MIMO antenna in both the E- and H-planes.

### 3.2. Measurement Results

**Figure 10:**
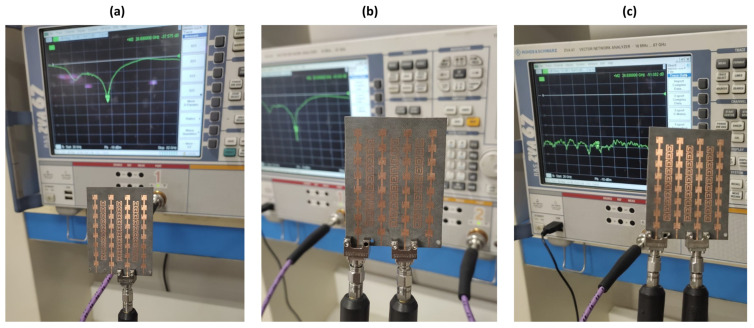
(**a**) Measurement setup of reflection coefficient of individual antenna port, (**b**) measured reflection coefficient of two ports, and (**c**) setup of measuring mutual coupling between each port pair.

A vector network analyzer (VNA) operating to 67 GHz was used to measure the scattering parameters of the antennas fabricated. The antennas under test were connected to the VNA through 1.85 mm end-launch connectors (Southwest Microwave Inc., Tempe, AZ, USA) as illustrated in Figure 10a. Quad-port MIMO antennas were prototyped and experimentally evaluated. Figure 10b shows setup for measuring individual reflection coefficient of each port (i.e., S_11_, S_22_, S_33_, and S_44_). Figure 10c displays the setup for measuring mutual coupling between each pair of antennas.

**Figure 11:**
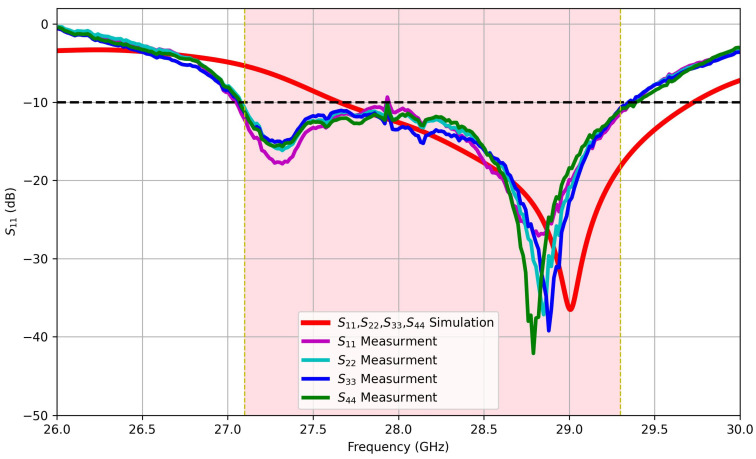
Measured and simulated reflection coefficients for each MIMO port, S_11_, S_22_, S_33_, and S_44_.

Figure 11 shows a comparison between measured and simulated reflection coefficients as the fabrication process requires a lot of specifications that should be taken into consideration to give the best results. There are some challenges facing the implementation of the proposed design that is listed below. The fabrication complexity is firstly concerned, as metamaterial unit cells often require high-precision manufacturing techniques such as photolithography or multilayer PCB fabrication, especially in the higher frequency spectrum at 28 GHz, which can introduce alignment errors and increase production costs. Additionally, fabrication tolerances leading to variation in the electrical properties of the proposed antenna and the MTM unit cell will vary in terms of the permittivity and permeability and will shift the operating frequency. Integration challenges also arise when implementing metamaterial structures in compact devices, as the MTM unit cell insertion must not affect the antenna radiation efficiency. Finally, the validation of the proposed design and accurate measurement require controlled testing environments, such as anechoic chambers, to ensure reliable mutual coupling reduction. Discrepancies between simulated and fabricated designs must be carefully analyzed to ensure consistency across MIMO designs. Addressing these challenges is essential for the successful realization of the proposed antenna structure in practical communication systems. The measured and simulated results exhibit generally good agreement within the specified frequency bands. However, a discrepancy of approximately 0.8 GHz is observed in the resonance frequency of the central band. This frequency shift could be attributed to several factors, including impedance mismatch, the effect of the SMA interface connector, environmental conditions such as temperature or humidity, and potential assumptions made during the modeling process that may not fully capture real-world conditions. Additionally, fabrication tolerances, such as slight variations in material properties or dimensional inaccuracies, may contribute to this deviation. Despite this offset, the measured bandwidths remain in close agreement with the simulated values, further indicating that the antenna’s performance in terms of bandwidth is consistent across the three frequency bands. This suggests that while the resonance frequency is slightly altered, the overall functionality and design objectives of the antenna are still achieved in the measured frequency range, confirming the robustness of the antenna’s performance. Measurement results show the reflection coefficients for each MIMO port (S_11_, S_22_, S_33_, and S_44_) against the predictions from simulation. The experimental results correlate well with theoretical predictions and thus prove that the design analysis is robust. It can also be seen from the measured results that there is a significant increase in the overall bandwidth across the investigated band, as well as a considerable decrease in the measured reflection coefficients, thus validating the effectiveness of these design improvements in general antenna performance. Findings reveal the essentiality of precise simulations together with thorough real-world experiments towards optimizing MIMO antenna characteristics for superior real-world functionality.

**Figure 12:**
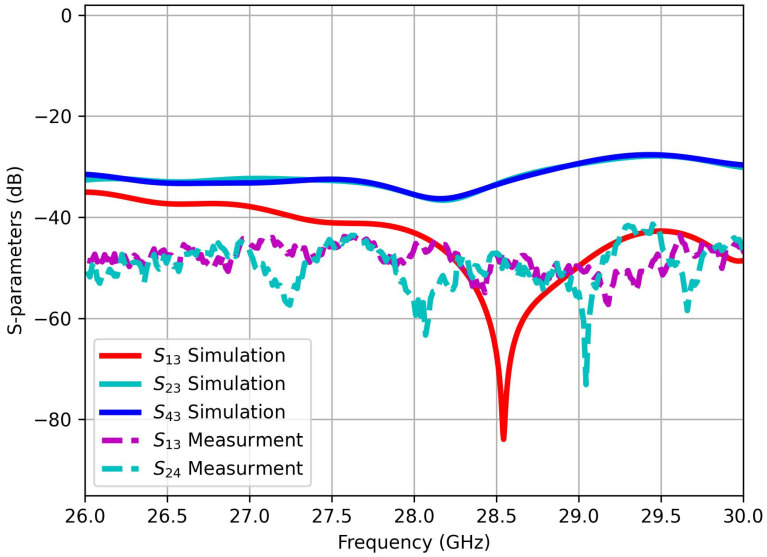
Simulated and measured S-parameters of 4-port MIMO antenna.

Figure 12 compares the simulated and measured S-parameters of the four-port MIMO antenna. The results demonstrate a strong correlation between the simulated and experimental data, indicating a significant reduction in mutual coupling among the MIMO ports. This alignment reflects a high degree of isolation across all three frequency bands, emphasizing the efficacy of the metamaterial design in improving MIMO isolation. The enhanced isolation minimizes inter-port interference, thereby optimizing antenna performance and contributing to improved reliability and efficiency in wireless communication systems.

Table 2 presents a comparison dataset compiling the latest reports that benchmark the proposed design. Indeed, the proposed design has produced a very competitive result, according to the various antenna metrics of bandwidth gain and isolation. The comparative analysis, however, indicates the superiority offered by using the MIMO antenna over other existing antenna designs in terms of increased bandwidth efficiency, gain, isolation specifications, and overall performance. By considering the proposed design in the light of recent advances, this evaluation demonstrates its effectiveness and great potential in ushering MIMO antenna technology into the next-generation wireless communication system.

Considerable coupling between elements in a MIMO configuration can be very detrimental to system performance because it tends to impend substantial surface wave interactions. Therefore, a deliberate attempt must be made to make it very possible to reduce interference or crosstalk between antenna elements. The results in this work were coupling reductions of −40 dB. This is good evidence to show that the undesirable coupling effects have been reduced, leading to better isolation and improved performance over tri-band operation because such significant mutual coupling reduction proves the effectiveness of the proposed design strategies for making MIMO antenna systems more reliable and efficient for wireless communication.

## 4. Conclusions

This study introduces an advanced design for a MIMO antenna engineered for operation in the 28 GHz frequency band. The MIMO antenna setup consists of four ports, featuring 1 × 8 series-fed arrays for each port, showing peak gain of 15.5 dBi and bandwidths spanning 2 GHz. The enhanced performance of this antenna design is a direct outcome of meticulously optimized antenna spacing and a decoupling strategy employing well-designed metamaterial cells, effectively reducing interference among antenna elements. The system demonstrates remarkably low mutual coupling, registering below −40 dB, with envelope correlation coefficients of 0.00010, diversity gains approaching 10 dB, and a channel loss capacity of 0.11 bit/s/Hz across the examined frequency range. Experimental assessments have validated these enhancements, establishing the proposed design as a promising candidate suitable for a millimeter-wave communication networks.

## Figures and Tables

**Table 1:** Antenna array dimension parameters.

Parameter	*W_mf_*	*L_mf_*	*W_f_*	*L_f_*	*W_p_*	*L_p_*	*W_s_*	*L_s_*	*W_g_*	*L_g_*	*W_gs_*	*L_gs_*
**Value (mm)**	0.6	6	0.5	4.16	3.72	3.61	12	65	11	59	0.5	6

**Table 2:** Comparing the achieved results of this design with those of other designs in terms of various evaluation metrics.

Reference No.	Operating Frequency (GHz)	No. of Ports	Dimensions (mm^2^)	Bandwidth (GHz)	Gain (dBi)	Isolation (dB)
[33]	28	4	30 × 35	4.1	8.3	22
[34]	28	4	32 × 8	2.6	7.5	25
[35]	28/38	2	55 × 110	1.07/1.43	7/8	27
[36]	28/38	2	7.5 × 8.8	1.23/1.06	6.6/5.86	20
[37]	28/39/48.7	2	10 × 16	1.8/6.2/3.8	9.5/11.5	17
This work	28.8	4	48 × 65	2.0	15	>40

## Data Availability

All data supporting the findings of this study are already included within the article.
